# In search of the last malaria cases: ethnographic methods for community and private-sector engagement in malaria elimination in Vietnam, Laos, and Cambodia

**DOI:** 10.1186/s12936-021-03903-y

**Published:** 2021-09-17

**Authors:** Yoriko Masunaga, Joan Muela Ribera, Thuan Thi Nguyen, Kemi Tesfazghi, Koen Peeters Grietens

**Affiliations:** 1 Socio-Ecological Health Research Unit, Department of Public Health, Institute of Tropical Medicine, Antwerp, Belgium; 2 Faculty of Social and Behavioural Science, Department of Sociology and Anthropology, University of Amsterdam, Amsterdam, The Netherlands; 3 PASS Suisse, Neuchâtel, Switzerland; 4 Universitat Rovira i Virgili, Tarragona, Spain; 5 National Institute of Malariology, Parasitology and Entomology, Hanoi, Vietnam; 6 Population Service International, Vientiane, Laos; 7 School of Tropical Medicine and Global Health, Nagasaki University, Nagasaki, Japan

**Keywords:** Malaria elimination, Ethnographic methods, Community engagement, Public–private mix, Greater Mekong Subregion

## Abstract

**Background:**

Despite significant strides made in reducing malaria morbidity and mortality in the Greater Mekong Subregion, malaria transmission continues amongst the most ‘hard-to-reach’, such as forest-goers and mobile and migrant populations, who face access obstacles to malaria diagnosis and treatment. As such, regional malaria elimination strategies endeavour to incorporate the private sector and local communities in improving surveillance and detection of the last malaria cases in remote forested areas. The question remains, however, whether such strategies can reach these hard-to-reach populations and effectively reduce their disproportionate burden of malaria. This paper evaluates the strategy of community and private sector engagement in a malaria elimination project in Vietnam, Laos, and Cambodia.

**Methods:**

Ethnographic research, incorporating in-depth interviews, participant observations with informal discussions, and group discussions were conducted in Bu Gia Map commune, Binh Phuc province of Vietnam; in Phouvong district, Attapeu province of Laos; and, in nine newly established and informal communities in the provinces of Mondul Kiri, Steung Treng, Kratie, Kampong Thom, and Prah Vihear of Cambodia.

**Results:**

Different types of factors limited or enhanced the effectiveness of the participatory approaches in the different settings. In Vietnam, inter-ethnic tensions and sensitivity around forest-work negatively affected local population’s health-seeking behaviour and consequent uptake of malaria testing and treatment. In Laos, the location of the project collaborative pharmacies in the district-centre were a mismatch for reaching hard-to-reach populations in remote villages. In Cambodia, the strategy of recruiting community malaria-workers, elected by the community members, did manage to reach the remote forested areas where people visited or stayed.

**Conclusions:**

‘Hard-to-reach’ populations remain hard to reach without proper research identifying the socio-economic-political environment and the key dynamics determining uptake in involved communities and populations. Solid implementation research with a strong ethnographic component is required to tailor malaria elimination strategies to local contexts.

## Background

The main malaria occurrence and transmission in Southeast Asia takes place in forests and forest fringes among specific risk groups, such as forest-goers and mobile and migrant populations who are commonly hard to reach [1–6]. In the Greater Mekong Subregion (GMS), connecting six countries through the Mekong River (Cambodia, China, Laos, Myanmar, Thailand, and Vietnam), malaria elimination strategies envision “a region free of malaria and the continual threat posed by anti-malarial drug resistance” by tailoring interventions to local contexts, improving surveillance, promoting a multisectoral approach, and addressing inequity in access to services for the most vulnerable and hard-to-reach populations [7].

Due to declining trends of malaria in the GMS over the past 15 years, more and more focused approaches are being employed to reach the last malaria cases mostly present along international borders, in remote and forested areas [8, 9], and among the ‘hard-to-reach’ populations, which often correlates with forest-goers and mobile and migrant populations [4, 7, 10]. The World Health Organization (WHO)’s *Strategy for Malaria Elimination in the Greater Mekong Subregion (2015–2030)* states that “elimination will not be achieved unless these population groups have access to malaria protection measures, early diagnosis and treatment” [7]. To scale up universal access to these preventive measures and quality case management, current malaria control practices employ free-of-charge rapid diagnostic tests (RDTs), artemisinin-based combination therapy, use of long-lasting insecticidal nets and indoor insecticide residual sprays [9]. In order to effectively implement these measures and deliver these services, especially to hard-to-reach populations, coordination and collaboration with health and non-health sectors, between health sectors, and with the communities has been promoted [7]. In particular, there is an increasing recognition of the need for private health sector involvement in malaria elimination efforts. Countries in the GMS have incorporated the use of private sector providers into their national strategies, i.e. public–private mix approach [7, 11], recognizing that people with febrile illness are most likely to seek care in the private sector (including informal care) [8, 12–14]. Additionally, as a high proportion of malaria cases are asymptomatic in the GMS [1, 3], and hence are unlikely to actively seek care [15], relying only on passive case detection at the public health facilities presents serious limitations for early diagnosis and treatment. In this way, the public–private mix approach attempts to increase access to early diagnosis and treatment, to improve malaria case reporting from the private sector, and to ensure the quality of service at both public and private sectors by standardizing care and regular monitoring [7, 16]. Moreover, as the WHO states that engaging community service providers, who are often volunteers from communities, is the best solution to provide services in remote areas [7], the GMS strategy of placing greater emphasis on community engagement strives to further increase this access to malaria control measures for these hard-to-reach populations.

However, despite these collaborative efforts and control measures, tackling malaria remains challenging due to regional complexities such as the uneven distribution of malaria cases, the biodiversity of vectors, heterogeneous human and vector behaviour [1, 5, 6, 9, 17, 18], the spread of drug resistance [6, 9, 19], remaining poor accessibility [5, 9], and high population mobility including cross-border movements into areas of higher malaria incidence [5, 9, 20]. Moreover, the uncritical application of standardized measures in diverse ethno-socio-cultural contexts ignores social parameters, generating pseudo outcomes, real in their adherence to standardized methods and fake in the applicability of the conclusions it draws for local populations [21, 22]. In the GMS, the inhabitants of forested areas originate from diverse ethnic minorities who respectively engage in specific economic activities. The activities and movements of these heterogeneous populations are further diversified in a constantly changing landscape due to deforestation and reforestation as a result of responding to global demands, such as acquiring raw materials (e.g. wood, rubber, gems, gold) and cultivating food (e.g. banana, cashew, coffee, cacao) [23], in order to sustain income. Moreover, these practices result in deforestation, which in turn is linked to greater malaria risk in GMS because it disrupts ecological conditions and creates favourable environment for malaria vectors (e.g. reduction in predators of mosquitoes and increased larval survivorship) [5, 6, 23–27].

Unfortunately, only a small number of studies have looked into the diversity among these populations and their respective forest-related economic activities, despite such insights being vital to adequately tailor elimination interventions [28]. Malaria control and elimination measures in the GMS are largely implemented based on the evidence that transmission continues amongst hard-to-reach populations widely (mis)categorized as forest-goers or mobile and migrant populations who face difficulties in accessing malaria diagnosis and treatment. These strategies are further based on the premise that incorporating the private sector and local communities in these interventions will bolster surveillance and detection of (the last) malaria cases in remote forested areas. The question remains whether such a strategy can effectively reach the ‘hard-to-reach’ and increase their intervention uptake. Previously, ethnographic studies have provided insights in relation to the uptake of malaria interventions, looking into the variability in the epidemiology, human behaviour, mobility patterns and the variety of social contexts [18, 20, 22, 29–36]. This paper calls to question the current premises guiding the GMS malaria elimination strategy by evaluating the manner in which communities and the private sector have been incorporated into the project “the Greater Mekong Subregion Elimination of Malaria through Surveillance (GEMS)” in Vietnam, Laos, and Cambodia.

## Methods

### PSI GEMS project

In order to contribute to the malaria elimination goal, Population Service International (PSI), a non-profit global health organization, launched the project “the Greater Mekong Subregion Elimination of Malaria through Surveillance (GEMS)” in Cambodia, Laos, Myanmar, and Vietnam. Between 2016 and 2019, GEMS engaged with a range of health care providers (i.e. clinics and pharmacies), community-based health providers (i.e. volunteer malaria-workers), and community-based outlets (i.e. grocery shops) to accelerate malaria case management, especially to foster active case detection striving to reach ‘hard-to-reach’ at-risk populations [37]. The GEMS provided these providers with RDTs, referral cards, and anti-malarial drugs, according to the characteristics of each national health system, and trained them to identify suspected cases and test, accurately treat or refer, and rapidly report cases to PSI. Data reported were incorporated into the national malaria control programme’s surveillance system. PSI tailored the project strategies to each country setting, including the roles of collaborative providers that were based on the requirements of the national health systems.

### Anthropological study

Anthropological study was carried out to evaluate the effectiveness of PSI’s interventions within the GEMS project, including community engagement and the public–private mix initiative in Vietnam, Laos, and Cambodia. Specifically, the study explored the factors influencing communities and private sector participation in the GEMS project (i.e. the actual uptake of services provided by the private formal/informal sector as well as service providers’ acceptance of their new role in malaria surveillance and case identification). Ultimately, the study aimed to identify bottlenecks and successes, which can be incorporated into future more tailored strategies. To this end, ethnographic methods were employed, which have been proven to be effective in assessing the health needs of ethnic minority groups, patient perceptions of medical services, and pluralistic concepts of illness and health [38]. Moreover, ethnography has been shown to help troubleshoot and improve interventions [39]. Among the techniques used in this study, participant observation and informal interviewing played a vital role as they served to lay bare the diversity and complexity of local populations, their perspectives, and reasons for action [40].

### Study sites

The study was conducted in the GEMS implementation sites in Vietnam, Laos, and Cambodia, based on the GEMS project progress.

#### Vietnam

The Bu Gia Map commune of Binh Phuc province in southeast Vietnam shares a border and forest with Cambodia. Bu Gia Map was purposefully selected due to the high self-reported malaria incidence among forest-goers (though without accurate incidence estimates) and the presence of multidrug-resistant malaria to treatment [41]. Moreover, there was a call by PSI to investigate the effectiveness of their newly piloted project strategy, which involved community malaria-workers and grocery-shops as non-health sector collaborators.

#### Laos

The Phouvong district of Attapeu province in southern Laos shares its border and forest with Vietnam and Cambodia. The Phouvong district was selected due to a dramatic increase in parasite incidence, accounting for 96% of the total cases in southern Laos (in 2014) with Attapeu province having the highest number of cases [2, 42]. Additionally, 68% of infections in 3 districts in Attapeu, including Phouvong, were asymptomatic [2]. Due to insufficient information on the population in remote villages and their mobility patterns and activities, anthropological research to provide in-depth information was required.

#### Cambodia

Nine newly established communities in forested areas not yet officially registered by the government were selected (i.e. Prey Khiev in Mondul Kiri province, Dai Ou Sav in Steung Treng province, Sen Chey and Ou Yeav in Kratie province, Kbal Ou Lang, Snorng On, and Rolous in Kampong Thom province, and Pkil and Cheal in Prah Vihear province). Due to their remoteness and unregistered status, they are not included in the national health scheme. These communities were selected to examine and evaluate the project implementation, which was executed after almost a yearlong community engagement process.

### Data collection

Data was collected in Vietnam in September, November, and December 2018, in Laos between May and August 2019, and in Cambodia from June to October 2019. Data was collected through in-depth interviews, informal conversations, group discussions, and participant observations during various stages of fieldwork, in which the anthropological study team repeatedly visited and stayed in study communities. The study team consisted of the international researchers and local research-assistants in each country. The researchers developed preliminary topic guides based on literature and GEMS reports in each country, and continuously refined and adapted the guides with research-assistants to address relevant emerging themes during the data collection process. Data collection continued until saturation (i.e. no more new findings emerged). In-depth interviews lasted approximately 40–60 min and group discussions 90–120 min, including translations of the conversations from local language(s) to English by the research-assistants. Most informal conversations took place during participant observations in villages, work sites (e.g. plantation sites, gold mining sites, dams), forest fringes and forests, health centres, pharmacies, and grocery-shops. Interviews were not audio-recorded due to the sensitivity of the topics discussed (e.g. ethnic tensions, illegal forest activities), but detailed notes were taken during or immediately after the conversations.

In each country, the local research-assistants contributed greatly to data collection: in Vietnam, the local researcher, who has extensive experience in qualitative studies in Vietnam, joined in the data collection process and played a key role in bridging researchers and the community. In Laos, a research-assistant from Attapeu who spoke one of the main ethnic languages in Phouvong district and who previously engaged in forest-work was able to conduct participant observations in remote villages and in the forest where access was difficult. In Cambodia, the GEMS project team and our research-assistants, who had gained substantial trust among the communities, helped to collect informal descriptive information as well as to carry out participant observation in remote areas.

### Sampling

Purposive, snowball, and maximum variation sampling techniques were used to identify respondents and study sites, in order to draw a systemic picture of the topic. In most cases, the GEMS project team introduced the anthropological study team to village heads, village health workers or PSI malaria-workers, whom then introduced other respondents relevant to the topic, such as ex-malaria patients. This was complemented with independent purposefully selected informants.

### Data analysis

Data analysis took place concurrent to data collection. Conversations were continuously analysed to test and confirm hypotheses, and to refine interview questions. The field-notes were organized digitally and cross-verified with all data collectors. Data were coded using NVivo (version 11) software.

### Ethical considerations

All respondents received an explanation about the research aim, confidentiality, anonymity and their right to withdraw from the study. Oral consent was obtained before each interview. Oral consent was preferred due to the high rates of illiteracy among the ethnic minority groups as well as to avoid sowing mistrust by obliging official signatures. Individual data were stored anonymously on a password-protected device only accessible by the researchers.

## Results

### Case study 1: inter-ethnic relations, Bu Gia Map commune, Vietnam

#### Study participants

A total of 71 in-depth interviews and participant observations with 40 informal conversations were conducted with public/private health staff, village health workers (VHWs), PSI malaria-worker, pharmacists, ex/current malaria patients, families of patients, forest plantation workers, forest security guards, pastors, and grocery-shop and cafe owners. Additionally, three group discussions were held with participants from the Stieng and M’nong ethnicities who trusted the study team and gave insightful information during the ethnographic study.

#### Local social and health system organization

Bu Gia Map commune consist of six villages traditionally inhabited by the Stieng and M’nong ethnic minority populations, with Tay, Nung, and Kinh (Vietnamese) ethnicities as migrant populations. The Stieng and M’nong are culturally distinct from the Kinh majority ethnic group in Vietnam as their customs and subsistence are traditionally dependent on the forest, such as following animist belief and engaging in slash-and-burn agriculture. Nowadays, most Stieng and M’nong in Bu Gia Map are Christian (either Catholic or Protestant) and are farmers engage in cashew, rubber, coffee, and pepper cultivation on plantations.

In Bu Gia Map, there is one commune health centre (CHC), a smattering of pharmacies and private clinics, and one VHW appointed in every village. Most villages are located along the paved road between the CHC and the forests, and the farthest village from the CHC is approximately 5 km. Almost all households in these villages have a motorbike and accessing the CHC is relatively easy. Malaria testing and treatment is provided free-of-charge at the public health facilities (i.e. CHC, provincial hospitals), and incentives are periodically offered to those who seek malaria care by various on-going malaria projects.

#### Multiple providers and services

There were several PSI collaborating pharmacies around the CHC, many grocery-shops near the villages and forest fringes, and one volunteer malaria-worker tasked with testing (RDTs), prompt referral of positive cases to the CHC or PSI collaborating private clinics in another communes (there were no PSI collaborating private clinics in Bu Gia Map at the time of the study), and reporting test results to the PSI. These collaborators were not assigned to give treatments. Pharmacies and grocery-shops were to administer RDTs to malaria-suspected customers (e.g. with fever). The malaria-worker who was selected by PSI from the ethnic minority group was expected to foster both passive and active case detection among ethnic minorities as well as among forest-goers. However, in reality there were impeding factors for these collaborators in Bu Gia Map to conduct the testing.

Pharmacy and grocery-shop workers commonly cited the close proximity to the CHC, where patients were afforded free-of-charge malaria care, and their own workload made taking on the additional un-incentivized task of testing arduous.

*“I treat fever but not malaria. ‘The malaria company’ (PSI) gave me RDTs but I haven’t used it because it’s not my area and it’s not bringing me any benefits. I have no time for it. There’s a CHC right there and the CHC has been working on malaria for a long time so I refer suspected patients to the CHC […] I agreed to collaborate with the company (PSI) because these ‘marketing people’ came many times to persuade me.”—Interview, Kinh pharmacist-1*.

An awkward tension between pharmacies and the CHC was also noted, as the CHC was receiving monetary incentives under the regional artemisinin initiative for detecting positive cases (as of end 2018). This signified fewer incentives for the CHC when positive cases were referred to elsewhere (i.e. PSI collaborating private clinics), and thus some pharmacies were hesitant to test for PSI.

*“The CHC gets incentives for detecting malaria and I’m under CHC’s supervision.”—Interview, Kinh pharmacist-2*.

None of the interviewed local respondents were aware of the malaria service provided at grocery-shops. Respondents expressed concern in consulting grocery-shops for malaria testing as shop-owners are not medical personnel, and moreover, they showed dissatisfaction in receiving only diagnosis.

*“She [shop owner] can test malaria? But who would go to the shop to check for malaria? The shop owner doesn’t even have a medical profile so nobody would go there for health concerns. People don’t want just testing, but treatment.”—Interview, M’nong woman*.

In Bu Gia Map, PSI had appointed a Stieng ‘forest-goer’ as the malaria-worker. However, this forest-goer happened to be the local forest protection officer who lived just a few houses away from the CHC. At the time of the study, the malaria-worker said he had conducted only a few tests and found one malaria case since he took this role. Most interviewed local residents or medical personnel did not know of him or his role as a malaria-worker. He explained why people did not visit him for malaria:

*“Some people might know about my role as [malaria-worker], but people who feel ill would go directly to CHC as it’s very close to my house. The main reason people don’t consult me about malaria issues is because I’m not a qualified health personnel.”—Interview, Malaria-worker*.

#### Why testing was under par?

The Stieng and the M’nong ethnic minorities who traditionally lived in the forest have since been resettled in government provided villages, with their traditional agricultural practices in the forest being banned due to strict forest protection measures. A new land management system redefined the boundary between villages, farming land and protected forest areas, and restricted the locals’ traditional forest-based subsistence and economic activities. As a result, many locals have been evicted from their traditional lands and even jailed.

*“There is a ticking bomb tension between us, the indigenous, and the local government. […] We still go to the forest when we need to because that’s how we’ve been living. But now such action is considered illegal.”—Interview, Stieng man*.

Inter-ethnic tensions as well as the sensitivity surrounding forest work have likewise shaped care-seeking behaviour among these ‘hard-to-reach’ populations. Specifically, local distrust of the Kinh by Stieng and M’nong minorities has manifest in the latter’s avoidance of Kinh-run institutions.

*“We prefer to have a trusted medical doctor like the one in [another commune] (Stieng). […] Currently medical personnel in our area only want us to be sick so that we would return to them many times.”—Discussion, Stieng and M’nong residents*.

Notably, all pharmacists and grocery-shop owners interviewed in Bu Gia Map were Kinh, further impeding malaria testing at these sites. Though the local malaria-worker was Stieng and could arguably serve as liaison between the local ethnic minorities and health institutions to promote testing, his occupation as a forest protection officer bred antagonism among many local forest-goers whose activities were officially deemed illegal. Moreover, in Vietnam, the public health sector widely covers populations notwithstanding the issue of inter-ethnic tension observed in Bu Gia Map, and there was little space for private and non-health sector service provision.

### Case study 2: navigation in remoteness, Phouvong district, Laos

#### Study participants

Participant observations along with 48 in-depth interviews, 158 informal conversations, and 18 group discussions with village heads, public health staff, VHWs, pharmacists, ex/current malaria patients, families of patients, farmers and plantation workers, forest workers, dam workers, shop owners and soldiers were carried out.

#### Local social and health system organization

There are 22 villages scattered in the Phouvong district: 11 villages are situated within 1–8 km of the district-centre, 5 villages 17–34 km away from the district-centre; and 6 villages are located in remote areas in the forest fringe or in mountains closer to Vietnam border (37–122 km from the district-centre). In the district-centre, there is Phouvong district hospital and 3 pharmacies. There are 5 health centres spread throughout the district, and 1–2 VHW(s) in most of the 22 villages.

The Brao ethnic minority is the predominant ethnic group in Phouvong district, followed by the Kayong and Sadang ethnic minorities. In the 1970s, these ethnic minority groups were officially resettled from their traditional villages in the forest to these 22 government-administered villages. Nevertheless, they continue to maintain a decided presence at their traditional forest lands. The Brao, Kayong and Sadang are traditionally animist (although this is changing especially among Sadang who are slowly converting to Christianity) and consult shamans, conduct rituals (e.g. offer rice, alcohol, animals to the spirits), and rely on herbal medicines for health.

#### Public–private mix and the village health workers

The PSI Laos strategy reinforced the public–private mix in which specific private health providers, pharmacies, and grocery-shops are also charged with testing and treating or referring malaria cases to the public health sector. The army also has a malaria testing site where PSI Laos had begun collaboration to synthesize the testing numbers and positive cases. In Phouvong, only 3 pharmacies located near the district hospital were participating in the public–private mix initiative. Specifically, the presumed advantage of pharmacy consultancy lies in its easy accessibility and low level of administrative paperwork when compared to public health counterparts.

*“Usually people go to the pharmacy to get medicine, then go to the hospital. So the pharmacy is the step between making an offering (to the spirits) and the hospital.”—Discussion, Brao residents*.

Additionally in Phouvong, VHWs, who are appointed by the government and the village head to deal with general health concerns, took on a more focused role as malaria-workers. However, a number of barriers to consulting VHWs were raised by local respondents, among which included the frequent lack of diagnostic and/or treatment tools at the VHWs’ disposal as well as the fact that VHWs had to meet their own work requirements in addition to their work as health providers.

*“Someone was sick with malaria, and she went to the VHW. She received malaria medicine without receiving RDT. […] The VHW is also working and it’s difficult to find her.”—Discussion, Lao, Brao, and Kayong residents*.

Moreover, VHWs becoming malaria-workers was perceived as more of a deterrent than an incentive to consult them as respondents considered this shift in functionality to limit VHWs’ competency in treating other illnesses.

*“The VHW can just test and give medicine for malaria but other illness he can’t treat. But we don’t know if the illness is malaria or not, that’s why we go to the health-centre.”—Interview, Brao man*.

#### Are the ‘hard-to-reach’ being reached?

The premise guiding collaboration with pharmacies was to establish another means by which malaria testing would be available. However in Phouvong district, the persistent barrier to maximal coverage remained the considerable distance between pharmacies and remote settlements that could be up to 122 km away by unpaved forested/mountainous roads. Closer pharmacists or clinics available to them were across the border in Vietnam, but where health care costs are higher. Residents in remote settlements frequently have to use both boats and motorbikes to cross rivers and make it through treacherous mountain passes. For non-residents (e.g. health staff from the district) who are not familiar with the forest-roads, passing forested and muddy paths is almost impossible especially during the rainy season. Personnel from the district hospital and health centres confirmed that they are unaware of the malaria situation in remote villages during the rainy (high malaria incidence) season because they cannot reach them.

*The road conditions to go to [remote] villages are really bad. In the rainy season only motorbikes can go but not cars or trucks. The road has a lot of clay and it is difficult to ride a motorbike there. One motorbike needs 2 people (to assist each other). In the rainy season there are a lot of land leeches on the way, and if we stop to relax on the road, land leeches will catch you.—Participant observation, Phuyang village, August 2019*.

The isolation of remote villages from accessing basic public health services is further reinforced by stereotypes and fears that these ethnic minorities have powerful shamans who can hurt outsiders.

*“Many people were working on malaria […], but after a staff from the WHO died of malaria, nobody wants to work on malaria. […] Some people said that the WHO staff who died was killed by the prayers of a shaman in [X village], because he had some villagers working on a project for him but he didn’t pay them because he was unhappy with their work. After that, no one wants to work on malaria, because they are afraid to be killed by a shaman.”—Informal conversation, Health staff*.

Ethnic minorities relying on shamanism, spiritual rituals, or herbal medicines are often perceived by the health care personnel as a cause of delay in seeking medical care. According to health staff, a “wait and see” attitude with initial uptake of herbal medicines along with the practice of spiritual rituals in which patients and their family members are banned from exiting their house for a few days leads to delay. However, a more complicated conjuncture of conditions (e.g. the state of the roads to a health facility; workload in the farm or forest; severity of symptoms; or availability of cash) guiding people’s choice for care was observed.

*“If I have some money, I will go to the health-centre, but if I don’t have money, I will make a sacrifice. (…) I will make a sacrifice first because I don’t have money but I do have chicken for making a sacrifice.”—Informal conversation, Brao woman*.

This narrative also changes depending on the remoteness of the village and its proximity to the health centres.

Huaykood—8 km from district-centre and nearby a health-centre:

*“When we feel ill, we go to the VHW to check. If [the result is] negative for malaria, we go to a health-centre, and if negative we go to district hospital. If it is still negative, we apologize and ask forgiveness from the spirit. […] If we don’t recover after the offering, we go back to the hospital.”—Interview, Brao man*.

Phuyang—122 km from district-centre:

*“First, we make a sacrifice, after that we treat ourselves with traditional medicine or herbs at the house, because while making a sacrifice, we cannot go outside (due to the ritual taboo). If the treatment in the house is not complete, we will go to the health-centre.”—Informal conversation, Sadang man*.

Despite the attempt to increase accessibility to testing and treatment of malaria in Phouvong, the structural obstacles barring access to these services—namely poor infrastructure and considerable distances to reaching the nearest biomedical health provider—remained as barriers to ultimately reach this goal.

### Case study 3: informal settlements, Cambodia

#### Study participants

There are new and informal communities in many provinces of Cambodia, mainly in forested and remote areas, that are yet to be officially registered by the government. In some of these communities, participant observations along with 128 in-depth interviews and 87 informal conversations were carried out with community heads, public and private health practitioners, PSI malaria-workers, ex/current-malaria patients, families of patients, Islamic leaders, monks, teachers, shop owners, mobile-shop-sellers, farmers, gold miners, mechanics, and plantation workers. Two group discussions were held in one community.

#### Local social and health system organization

Prey Khiev in Mondul Kiri province, Dai Ou Sav in Steung Treng province, Sen Chey and Ou Yeav in Kratie province, Kbal Ou Lang, Snorng On, and Rolous in Kampong Thom province, Pkil and Cheal in Prah Vihear province are new communities located along the forest fringe or inside the forest, in remote areas far from the main roads or towns in each province. Some communities are located geographically close to each other, though access remains difficult due to difficult conditions of the forest paths. These communities are not yet formally registered by the government, mainly due to insufficient household numbers needed to fulfil a registration requirement as a formal community (150 households).

The residents of these settlements are mainly Khmer and Cham (also called Khmer-Muslim) ethnicities, who have migrated to the new communities from different provinces looking for land to live and cultivate on. Some residents were still in the process of clearing the forest or building houses, and would move back and forth between their homelands (to gain income by working on the farm) and the new community (to clear the land or build a house). Some households living in the communities had already settled in the area in the 1970s to escape the Khmer-Rouge. As these communities remain officially unregistered, they lack basic services such as schools and health care. Notably, no VHW had been appointed. Therefore, residents in these unregistered remote communities struggle reaching health care service.

*One evening in the Snorng On community, a woman had seizure and collapsed. Many people from the community went to check on her condition. She was unconscious. They discussed bringing her to Tum Rieng (a nearby town) with many health facilities. The motorbike was difficult to bring the patient who was unconscious. The walking tractor was too slow with these bad road conditions. The last option was to carry her in the hammock. Soon the decision was made, neighbours brought hammocks and put the patient in them. Almost every household of Snorng On was there to help, and about 100 people walked/ran so that they could rotate carrying the patient every 300-500 m. Some motorbikes accompanied to light the way with the bike lamp. Other motorbikes led the way praying to Nak Ta (the guardian spirit) along the way with rice and incense. Others put grasses on the patient’s head hoping her condition would get better. They all ran about 13 km, taking them 2.5 h, until they reached close to the main road and found a car that took the patient to Tum Rieng.—Participant Observation, Snorng On, July 2019*.

In this setting, self-treatment or informal health care providers have gained popularity. Informal health providers are community members who diagnose and treat illness, including malaria, with a variety of medicines, injections, and infusions, and offer free-of-charge shelter and food during treatment. Often, informal health providers were combat medics for the Khmer-Rouge, trained to care for soldiers, but without having an official medical certificate. Community members are usually aware that informal health providers lack official medical certification; nevertheless, they visit these providers as their care is perceived to be good. Most importantly, these providers are the most easily available and accessible, even though malaria diagnosis and treatment can cost around 300,000KHR (± 75USD) or more, while it is free-of-charge at public health facilities.

#### Community elected malaria-workers

Due to the 2018 ban on the private sector malaria testing and treatment in Cambodia, there were no private health care providers collaborating with the PSI. Consequently, PSI Cambodia focused on collaboration with community malaria-workers who are trained to conduct RDTs, treat positive cases, refer cases when necessary, and report the number of RDTs and positive cases to PSI. PSI Cambodia strategy was established through a yearlong community engagement process and based on the learnings from the project implementation and evaluation in Vietnam.

Malaria-workers were appointed through a PSI supported community engagement process whereby community members selected candidates and then elected their representative malaria-workers. This election process itself served as a proclamation in a community about newly appointed malaria-worker(s) and their new role.

*“I was in the [malaria-worker] election process, that’s why I know (about malaria-worker). I’m happy with the result because now we don’t have to travel too far.”—Interview, Cham man*.

Being recognized by their community in their new function seemed to more greatly encourage malaria-workers to conduct testing, particularly active case detection, which requires visiting households in remote areas or the deep-forest.

*“I can go and test because I know people. I go to worksites [in remote areas] every 2–3 days [for active case detection duty]. I bring RDT and medicines there, and I test people who suspect malaria. They live in the hut and road condition there is bushy. […] People there go to [a] health-centre, but they need a boat to get there and takes about 2 hours.”—Interview, Malaria-worker*.

In addition to malaria care, the PSI sponsors malaria-workers to provide oral rehydration solution Orasel and condoms (except in Muslim populations) in an effort to widen their services to beyond that of malaria. Orasel was welcomed by recipients who perceived the vitamin to help with malaria treatment and who otherwise would have gone to a private clinic or an informal health care provider to similarly receive an infusion to “regain energy” for a quick recovery. It also motivated people to visit malaria-workers for consultations beyond malaria care.

*“My son had diarrhoea so we went to [the malaria-worker]. […] I got [orasel] for diarrhoea from him […].”—Interview, Khmer woman*.

#### What was recognized in unrecognized communities?

Appointing malaria-workers through a system of election where community members were actively involved in the transparent appointment of peers for this new function was successful. This process more readily fostered acceptance and acknowledgement of the malaria-workers and their new role in the community.

*“There are several points [why he was chosen as malaria-worker]. He is friendly and kind. He supports others, and he doesn’t look down or pity other people.”—Interview, Cham woman*.

Malaria-workers who are residents of remote and unregistered community understood well the complexity of activity and mobility patterns that are due to: (i) new settlers fluctuating between their homeland and the new community; (ii) engagement in multiple diverse activities to sustain income (e.g. hunting, fishing, gold mining, logging, forest-vegetable collection, and plantation work); and (iii) travelling to access basic services such as health care, markets, centres of education in nearby (but difficult to reach) towns. All of these activities and movements involve forest-going to some extent or another, as all communities are located at the forest fringes and/or in the forest. Most adults in these communities, both men and women, frequently go to the forest or deep-forest either for a short period of time (i.e. 1–7 days) or medium-long period of time (i.e. 1 week up to a few months) for economic and self-subsistence activities (Table 1). Malaria-workers have social ties with people and they share information about forest-going and, therefore, they are aware of the profile and schedule of people going to the forest/deep-forest, which allowed them to more easily reach the ‘hard-to-reach’.

**Table 1:** Example of diversification of activities in Cambodia

Activities	With	No. of stay	Sleep	Protection	Season
Plantation (around houses)	Family, labourers	NA	House	Bed-net	Rain
Plantation (owned by company)	Family, friends	Weeks– months	Company’s hut	Bed-net, Hammock-net	All
Mountain rice-field	Family	A day	NA	NA	Rain
Collect vegetables	Alone, family, friends	Daytime	NA	NA	All
Cutting woods	Family, friends	2–10 nights	Outside/simple hut	Hammock-net or nothing	All
Gold mining	Family, labourers	A day– months Months	Hut Hut	Bed-nets Bed-nets, Hammock-net or nothing	Rain
Hunting	Alone, friends	A day Hunt at night Few nights	NA NA Outside	NA NA Hammock-net or nothing	All
Fishing mountain stream	Alone, friends	A day 2–3 nights	NA Simple hut	NA Hammock-net or nothing	All

Importantly, the notion of ‘forest’ was not static. While outsiders (e.g. malaria projects, health staff, officials) perceived these communities to be situated in the forest, local residents differentiated the village, the cleared areas in the forest for plantation work, the forest, and the deep-forest. Moreover, this differentiation was also contingent on how long ago the respective residents had settled in the area. This heterogeneous distinction of ‘forest’ similarly manifested in heterogeneous malaria risk perceptions.

*R1: “The ones who go to the forest have more risk of malaria: gold miners and forest-goers.”*

*R2: “Not only in the forest, but here in the village you also have risk. Because here is in the forest.”—Discussion, Khmer residents*.

Additionally in Cambodia case, community members’ familiarity of consulting non-medical informal health care providers supported malaria-workers not being professional medical personnel. Furthermore, people’s preference for receiving ‘energy’ infusions (replaced by Orasel) worked positively for malaria-workers perceived work capacity.

## Discussion

In an effort to eliminate malaria in the Greater Mekong Subregion (GMS), the regional elimination strategy focuses on identifying and effectively treating every malaria case [7]. As malaria numbers decline, the last malaria cases have become concentrated among ‘hard-to-reach’ populations in remote and forested areas. Engaging communities and private providers was, therefore, included as a strategy to increase chances of identification and treatment of malaria cases among these vulnerable and elusive populations [7, 10]. Namely, the assumptions that community members’ familiarity with their community made them best equipped to actively detect malaria among their ‘hard-to-reach’ counterparts; and that the prevalence and popularity of private service providers made them ideally positioned to provide screening to their customers. In this section, the accuracy of these premises guiding, among other interventions, the GEMS project implemented in Vietnam, Laos, and Cambodia is discussed, as well as the factors that hamstringed the current GMS malaria elimination strategy.

### Blind premises: community and private-sector engagement

Engaging the communities and the private sector in malaria elimination strategies has the potential to increase access to malaria testing and treatment, as well as to boost people’s acceptability of interventions. For an effective community and private-sector engagement, capturing context-specific enablers and obstacles becomes crucial [43]. Case in point, the Vietnamese public–private mix and community engagement strategy faced challenges due to the sensitivity of a setting that was rife with inter-ethnic tension and mistrust concerning the very research being carried out. Firstly, the inter-ethnic tensions manifested in the fact that the local population, predominantly disenfranchised ethnic minority Stieng and M’nong, were less likely or willing to seek testing and/or treatment at Kinh-run institutions. Secondly, doubt in receiving health care by non-medical personnel reduced people’s willingness to seek care at the grocery-shops or malaria-workers, partly due to the extensive coverage of public health care provision at the primary level. Thirdly, the sensitivity around forest activities in protected forests fuelled antagonism between the malaria-worker in Bu Gia Map—who worked as a forest protection officer—and the residents whose subsistence depended on (illegal) forest activity. The malaria-worker was selected among the ‘forest-goers’, defined by PSI as “who have stayed in the forest or on a farm or plantation for at least one night in the last 3 months” [44]. This generalized categorization of ‘forest-goers’ lacked the diversity and the conflicting power dynamics among this population. Understanding these dynamics prior to the project implementation would have allowed a more precise representation of the forest-goers the project thrived to reach. Unfortunately, in this case, the malaria-worker represented the ‘power’ rather than the local community, which risked to widen the gap within the community [45]. It takes an active effort to involve marginalized populations as often opportunities to represent and speak are mediated or manipulated by the people who provide them in the first place [46, 47]. On the contrary, PSI Cambodia’s approach showed that adequate research, based on lessons learnt from Vietnam, prior to strategy implementation can lead to increased success for project uptake. PSI Cambodia conducted formative research in which the project team visited each community and households in remote areas, and realized the pivotal importance of electing malaria-workers locally and to equipping them with vitamin supplements in addition to malaria care. Various factors (e.g. health system provisions, geographical environment, ethnic-relations) influence the success and limitations of strategy implementation, and the study findings suggest that robust investigation prior to implementation reduces these implementation blind spots.

### Deconstructing access issues

Only by first understanding the realities determining access to health services among targeted populations can appropriately strategies for communities and private sector involvement be developed. The study in Laos and Cambodia revealed the physical challenges in accessing health services by people living in remote areas, especially during the rainy season when the workload is high on farms or in gold mines, cash availability is low, road conditions are treacherous, and malaria is most prevalent. Facing multiple hurdles, people must navigate a fluid environment [48] assessing various conditions (e.g. weather, road conditions, symptom severity, and available cash and tools) before pursuing care. Under these circumstances, people’s choice of care depends largely on what is available and accessible [29], which in many instances may include traditional rituals, herbal medicine, or informal health care providers. Notably, this geographical access problem blocks not only people in remote areas from accessing biomedical care but also health providers from reaching these communities.

However, access to adequate health care services is not necessarily limited to geographic barriers [49]. As seen in Vietnam, inter-ethnic tension influenced people’s choice of care. For instance, ethnic minority residents often opted to visit a Stieng ethnic minority private doctor in another commune to avoid Kinh doctors at the local health centre. In Laos, isolation from health services reinforced the use of shaman and traditional rituals as a last resort, further reinforcing the myth and fear of ethnic minority sorcerers, and making biomedical health providers reticent to actively reach these populations. In this sense, ‘hard-to-reach’ populations are not defined by geographic criteria only, but by a complex conjuncture of contextual factors [50]. However, failure to be aware of these key dynamics results in the ‘hard-to-reach’ remaining hard to reach.

### Generalizing the ungeneralizable

The application of a standardized strategy in various distinct settings undermines the goal of reaching and treating the last remaining malaria cases. In fact, a number of malaria-related studies carried out in the GMS have called for tailored approaches and strategies [8, 10, 22, 51], appropriateness of interventions for local social and environmental conditions [30], and redefined the groups of people at-risk to showcase this variety [52]. However, when it comes to interventions, diversity and complexity continue to be ignored while heterogeneous settings are forced into simplified over-standardized interventions.

An example of such generalization is people’s relation to the forest when categorizing “malaria risk among forest-goers”. As seen in Cambodia, for instance, the significance and perception of the forest differs from group to group. The notion of ‘village’ or ‘forest’ can change depending on the resident (i.e. settled resident or new-comer) and these different perspectives have implications for risk perception and the uptake of protective measures. Needless to say, forest-going is not a homogeneous act but entails various activities and mobilities. Furthermore, different mobility types and patterns show different vulnerability and risk [22], as well as (in)effectiveness of protective measures [30, 32]. When malaria control strategies emphasize the idea of “malaria risk in the *forest*” or that “*forest-goers* are at risk” without further clarification of the local understanding of places, spaces, people, and mobility, it can produce an oversimplified statement such as “malaria risk is high among forest-goers” and possibly lead to misguided premises and mistargeted strategy insufficient to realize the malaria elimination goal. Flexible strategies, such as ethnographic methods, to adapt to the rapidly changing environment (e.g. deforestation) that can impact malaria transmission patterns and at-risk populations will prove essential [27, 53]. Moreover, in-depth ethnographic research into different notions of the forest, various activities and mobility linked to the exposure to malaria is required [31, 33, 52]. This would avoid strategies such as tracking and recording forest-goers and their movements for surveillance and case management as this is highly sensitive and confrontational in a setting where forest-going activities are illegal but the basis of ethnic minorities’ culture, social organization and subsistence persist. Any research endeavours must first be well versed in these complex dynamics and avoid generalization and pseudo outcomes [21]. Ethnographic methods help to optimize appropriateness of interventions [30] and navigate malaria elimination effort in greater context sensitivity that can contribute to more effective and community accepted interventions.

### Study limitations

Formal introduction of the study team to the communities by the local official bodies (e.g. national malaria control programme) possibly caused hesitation and response bias from local communities. Moreover, as community members considered the researchers to be a part of the PSI, the project implementer, this might have initially influenced people’s response. The repeated and prolonged nature of the field stays and ethnographic study with the predominance of informal data collection methods allowed the respondents to overcome these initial barriers. The study findings present a limited part and point in time of the GEMS implementations, and consequently, unable to evaluate the implementation at all stages of the project.

## Conclusions

This paper questioned *a priori* demand and premise of engaging the communities and the private sector in order to accelerate malaria diagnosis and treatment among ‘hard-to-reach’ populations, and moreover argued that the laudable attempts to provide context-specific implementation have not sufficiently recognized ethnographic evidence. This study reaffirmed that the use of ethnographic research has helped to identify diversity among forest-goers with greater implications for malaria risk, identify physical, structural and social barriers and facilitators to accessing health care by vulnerable groups, as well as identify actual flaws and strengths in the current Greater Mekong Subregion strategy for malaria elimination. In addition to epidemiological inputs to tailor interventions, robust implementation research with strong anthropological insights for contextualization is required (see [54]). The ‘hard-to-reach’ will remain hard to reach in the absence of proper investigation and localized research to help halt the blind implementation of universalistic malaria elimination strategies on contextually divergent settings that ultimately do more harm than good.

## Data Availability

The datasets generated and/or analysed during the current study are not publicly available because participants did not consent to have their full transcripts made publicly available. However, the NVivo database with excerpts of the transcripts relevant to the study is available from the corresponding author on reasonable request.

## Abbreviations

GMS: Greater Mekong Subregion
WHO: World Health Organization
RDTs: Rapid Diagnostic Tests
GEMS: Greater Mekong Subregion Elimination of Malaria through Surveillance
PSI: Population Service International
VHWs: Village Health Workers
CHC: Commune Health Centre
